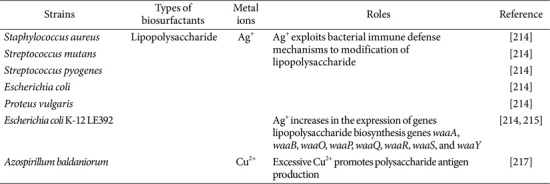# Review on Current Research on Biosynthesis of Biosurfactants and the Regulation Influenced by Metal Ions

**DOI:** 10.4014/jmb.2503.03031

**Published:** 2025-08-18

**Authors:** Yuchen Wang, Shan Qiao, Yongwu Niu

**Affiliations:** 1 College of Food Science and Technology, Henan University of Technology, Zhengzhou 450001, P.R. China; 2 National Engineering Research Center for Wheat & Corn Further Processing, Zhengzhou 450001, P.R. China; 3 Food Laboratory of Zhongyuan, Luohe, Henan 462300, P.R. China

**Keywords:** Biosurfactant, biosynthesis pathway, metal ions, regulation

## Abstract

Biosurfactants, amphiphilic secondary metabolites mainly produced by microorganisms, can be categorized into five groups according to their chemical structure and source: glycolipids, lipopeptides, phospholipids, polymeric biosurfactants, and particulate surfactants. The hydrophobic segments of biosurfactants typically contain fatty acids of varying chain lengths, while their hydrophilic portions display a wide range of diversity. Diverse biosurfactants have distinct metabolic pathways. Glycolipids are usually associated with glycolysis/gluconeogenesis and fatty acid metabolism, while lipopeptides are closely connected to non-ribosomal peptide synthetase. Particulate biosurfactants are formed through the anabolic pathway of phospholipids, with amino acid metabolism and carbohydrate metabolism being crucial components in the process of creating polar head groups. Metal ions are critical for either directly influencing strain growth or governing genes connected to biosurfactants production. This review explores the synthesis pathways of various biosurfactants and examines the influence of different metal ions on their production.

## Introduction

Biosurfactants are unique microbial secondary metabolites with notable surface activity [1]. These compounds are known for their amphiphilic structure, containing both hydrophilic and lipophilic groups [2], making them suitable for various industrial applications including bioremediation [3], pharmaceuticals [4], cosmetics [5], food processing [6], oil extraction [7], and many others. Global surfactant production surpassed 4.3 million tons, with a market valuation of 45.18 billion USD in the year 2023. The market is projected to expand to 69.13 billion USD in 2032, demonstrating a consistent annual growth trend thereafter [8]. In contrast to synthetic surfactants, which can be harmful to the environment due to their poor biodegradability and potential soil contamination [9-11], biosurfactants provide a more eco-friendly alternative. Due to their low toxicity [12], high biodegradability [13], excellent environmental compatibility [14], and ability to be synthesized from renewable raw materials [15], biosurfactants are commonly utilized in various industries. They serve as additives in cleaning products and laundry formulations [16], as emulsion stabilizers in the food and pharmaceutical sectors [17], and as cell repair and antioxidant components in cosmetics [18].

**Fig. 1:**
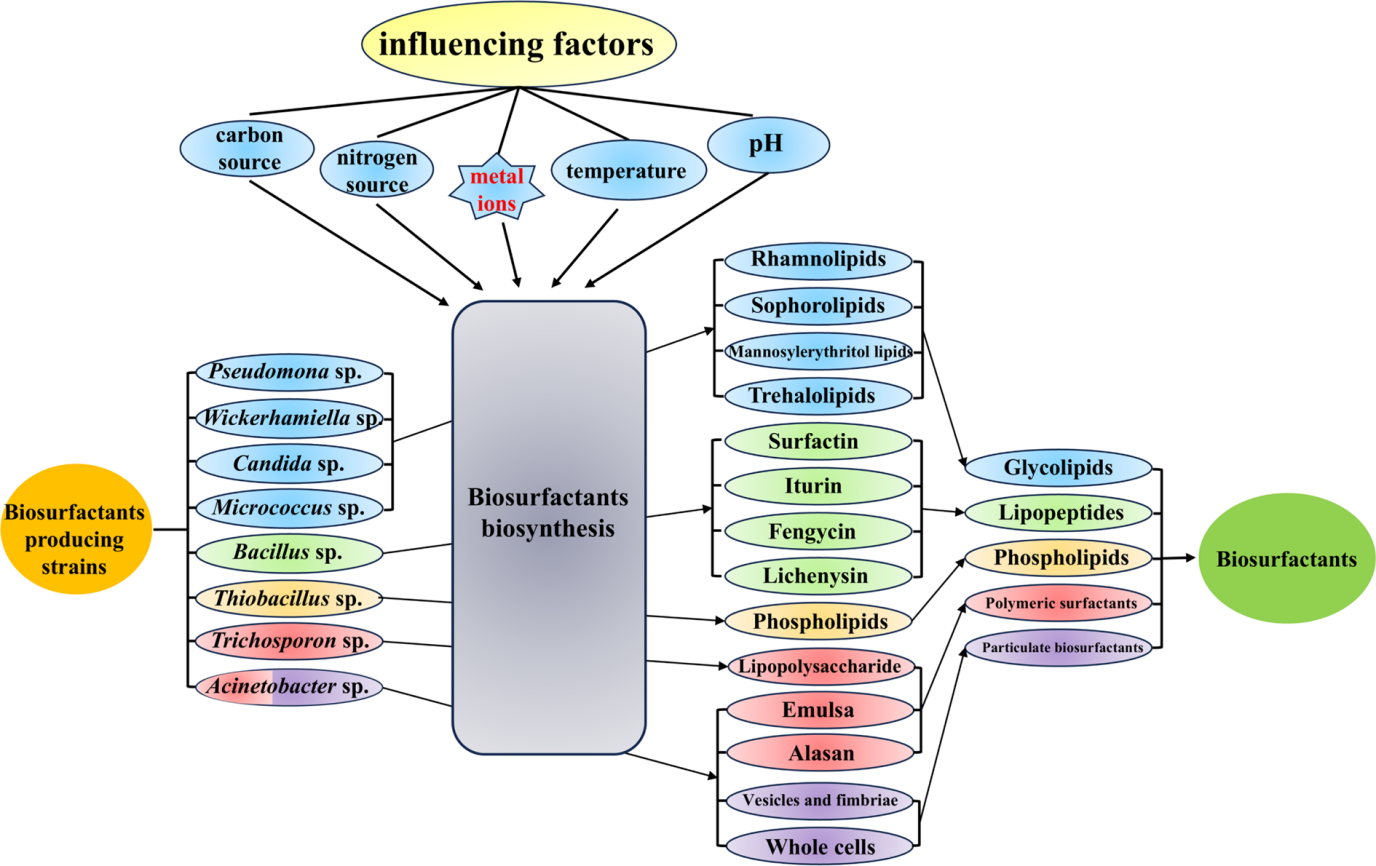
Source, classification, and influencing factors of biosurfactants.

Biosurfactants can be classified into five main groups based on their chemical structure: glycolipids, lipopeptides, phospholipids, polymeric biosurfactants, and particulate surfactants. Glycolipids are predominantly synthesized by *Pseudomonas* sp. [19], *Wickerhamiella* sp. [20], *Candida* sp. [21], and *Mycobacterium* sp. [22]. Lipopeptides are primarily produced by *Bacillus* sp. [23], while phospholipids are chiefly synthesized by *Thiobacillus* sp. [24]. Additionally, polymeric surfactants and particulate biosurfactants are mainly generated by *Trichosporon* sp. and *Acinetobacter* sp. [25]. The biosynthetic pathways for different types of biosurfactants exhibit significant variation, and the fermentation process for biosurfactant production can be influenced by various culture conditions, including carbon source, nitrogen source, pH, temperature, metal ion concentration, feeding regimen, and more. The source, classification and influencing factors of biosurfactants are shown in Fig. 1. In industrial production, certain metal ions often accumulate in fermentation media due to factors such as the composition of fermentation equipment, fermentation water, and raw materials. These ions serve dual roles: they act as essential nutrients aiding in the synthesis of specific enzymes in microorganisms, functioning either directly as coenzymes or indirectly by competing for enzyme active sites alongside substrates. This interaction helps to maintain enzyme activity and stability [26]. Recent studies have confirmed that metal ions such as Mg^2+^, Ca^2+^, Fe^2+^, and Mn^2+^ play a regulatory role in the biosurfactant synthesis of strains such as *Pseudomonas aeruginosa* and *Bacillus subtilis* [27-29]. However, most current research only looks at how metal ions affect surfactant production or structure at a macroscopic level, without delving into the underlying regulatory mechanisms. This review seeks to gather information on the biosynthetic pathways of different biosurfactants and shed light on the regulatory functions of various metal ions in biosurfactant synthesis.

## Biosynthesis of Biosurfactants

### Biosynthesis of Glycolipids

Glycolipids are found widely in nature and serve as important active substances that are crucial for facilitating the transmembrane transport and energy transfer processes of substances in organisms. Glycolipids, the most prevalent type of biosurfactants, are composed of hydrophobic long-chain fatty acids (aliphatic, hydroxylated, or unsaturated) and hydrophilic carbohydrate components (glucose, trehalose, mannose, galactose, sophorose, and rhamnose) [30 31]. The long-chain fatty acids and carbohydrates in the glycolipid components are produced through de novo synthesis, both directly sourced from the carbon in the medium [32]. The biosynthetic pathways of rhamnolipids, sophorolipids, mannosylerythritol lipids (MELs), and trehaloselipids in glycolipid biosurfactants have been extensively researched.

**Biosynthesis of rhamnolipids.** Rhamnolipids are natural glycolipid biosurfactants produced mainly by β- and *γ-Proteobacteria* [33]. Structurally, they are composed of rhamnosyl and β-hydroxy fatty acids linked through glycosidic and ester bonds [34]. Rhamnolipids are categorized into mono-RLs and di-RLs based on the number of rhamnosyl groups. These compounds are used in industries such as laundry detergents, cosmetics, food, and medicine [35] for their emulsifying properties, non-toxicity, and antibacterial effects [36]. Commercial rhamnolipids are primarily derived from secondary metabolites produced by *P. aeruginosa* in secondary fermentation [37].

**Fig. 2:**
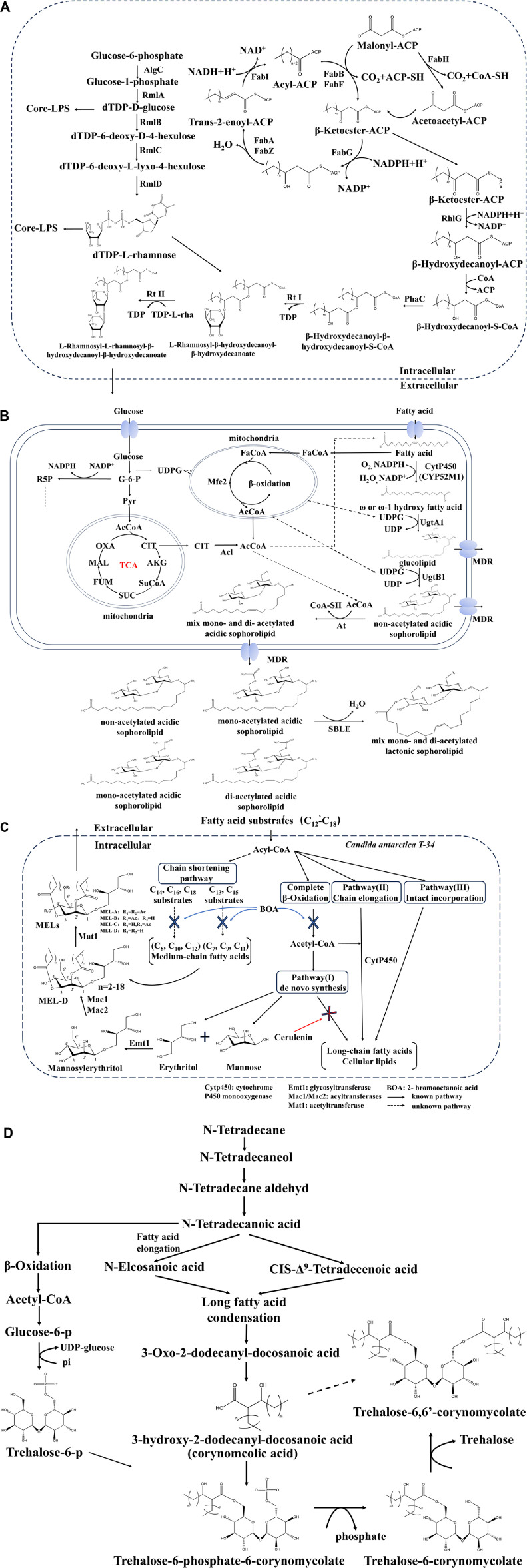
The biosynthesis pathway of rhamnolipids (A) sophorolipids (B) mannosylerythritol lipids (C) and trehalolipids (D).

The biosynthesis of deoxy-thymidine-diphospho-L-rhamnose (dTDP-rhamnose) consists of four steps starting from α-D-glucose-1-phosphate and thymidine triphosphate (TTP), passing through three intermediates before reaching the final product [38-41], as depicted in Fig. 2A. The four reactions are catalyzed in sequence by enzymes RmlA (α-D-glucose-1-phosphate thymidine acyltransferase), RmlB (dTDP-D-glucose-4,6-dehydratase), RmlC (dTDP-4-keto-6-deoxyglucose-3,5-epimerase), and RmlD (dTDP-6-deoxy-L-lyxo-4-hexulose reductase). The initiation of the β-hydroxy fatty acid biosynthesis cycle is carried out by 3-oxoacyl-[acyl-carrier-protein] synthase III (FabH), which converts acetyl-CoA and malonyl-ACP into acetyl-ACP. Subsequently, beta-ketoacyl-ACP synthase I (FabB) and beta-ketoacyl-acyl carrier protein synthase II (FabF) catalyze the condensation of malonyl-ACP and acyl-ACP. 3-oxoacyl-[acyl-carrier-protein] reductase (FabG) then reduces beta-ketolipids to beta-hydroxyacyl-ACP, followed by dehydration of beta-hydroxyacyl-ACP to form trans-2-enoyl-ACP by either beta-hydroxydecanoyl-ACP dehydrase (FabA) or 3-hydroxyacyl dehydratase (FabZ). To complete the process, trans-2-alkenyl-ACP is converted to acyl-ACP with the help of enoyl-[acyl-carrier-protein] reductases (FabI) in the final step [42]. After acyl-ACP is converted into β-hydroxydecanoyl-β-hydroxydecanoyl-S-CoA by β-ketoacyl reductase (RhlG) and polyhydroxyalkanoate synthase (PhaC), the interaction between dTDP-rhamnosyl and β-hydroxydecanoyl-β-hydroxydecanoyl-S-CoA is controlled by rhamnosyltransferase I (Rt I) and rhamnosyltransferase II (Rt II). Rt I is composed of two polypeptides encoded by *rhlA* and *rhlB*. It catalyzes monorhamnolipid formation from TDP-rhamnosyl and acyl-CoA. Rt II is a single protein encoded by *rhlC*, which is similar to rhamnosyltransferases in LPS biosynthesis. It converts monorhamnolipid and another TDP-rhamnosyl into di-rhamnolipid [35].

**Biosynthesis of sophorolipids.** Sophorolipids are a type of glycolipid biosurfactant produced by non-pathogenic yeasts, including *Candida apicola*, *Torulopsis gropengiesseri*, *Torulopsis bombicola*, and *Candida bogoriensis* [43-46]. Sophorolipids are known for their biodegradability [47], antibacterial activity [48], and environmental friendliness [49]. They have a wide range of applications in industries such as petroleum extraction, cosmetics, and medicine [50]. Naturally occurring sophorolipids consist of a mixture of different molecular structures with hydrophobic and hydrophilic components [51]. The hydrophobic part typically contains hydroxylated fatty acids, often located at the end or near the end of the chain, while the hydrophilic part is a sophoroglycoside made up of two glucose molecules linked by a β-1,2 glycosidic bond. Structural differences among sophorolipids include the position (6' or 6) and degree (none, mono-, or di-) of acetylation on the sophorose unit, the length of the carbon chain (usually C18), the site of hydroxylation (ω or ω-1) on the fatty acid, the level of unsaturation (number of double bonds), and the presence of a molecular lactone [52]. Sophorolipids can be classified into lactone sophorolipids and acid sophorolipids based on whether they have a free hydroxyl fatty acid tail. In lactone sophorolipids, a lactone bond forms between the carboxyl group of the hydrophobic fatty acid side chain and the 4' hydroxyl group of the hydrophilic sophorolipid [49, 52-55].

The biosynthetic pathway of sophorolipids involves fatty acid terminal hydroxylation, uridine diphosphate (UDP) - glucosyltransferase activity, hydroxyl acetylation, and the lactonization of sophorolipid ligands, as shown in Fig. 2B. These processes are mainly carried out by enzymes. such as cytochrome CYP52M1[56-58], UDP-glucosyltransferases A1 and B1 [59, 60], acetyltransferase [61], lactonase LIP1 [62], and sophorolipids transporter protein MDR [58]. The biosynthesis of sophorolipids begins with the hydroxylation of the ends of long-chain fatty acids. Fatty acids undergo catalysis by cytochrome P450 monooxygenase CYP52M1 to form ω-/ω-1 hydroxyl fatty acids. UDP-glucosyltransferase A1 (UgtA1) then attaches UDP-glucose to the hydroxyl group, forming a glycosidic bond. UDP-glucosyltransferase B1 (UgtB1) further extends this process by adding another UDP-glucose molecule to the glucose residue, creating long-chain ring-opening acid sophorolipids. Moreover, fatty acids are transformed into acetyl-CoA by peroxisomal β-oxidizing multifunctional enzyme type 2 (Mfe2). The acid sophorolipids are then transformed into acetylated sophorolipids and lactone sophorolipids by the enzymes acetyltransferase (At) and lactonase (Sble). The biosynthesis of sophorolipids is complex and interconnected with fatty acid metabolism, glycolysis, and gluconeogenesis [49, 63]. The acid and lactone forms of sophorolipids are eventually transported extracellularly by the MDR transporter [58].

Sophorolipids mainly obtain their fatty acid side chains from two sources: through the de novo synthesis of fatty acids using acetyl-CoA within the cell, and by directly taking up and transporting them from hydrophobic substrates in the medium, such as triglycerides, fatty acid esters, and hydrocarbons. Research conducted by Inoue [64] has shown that the yeast *C. bomicola* can produce sophorolipids when grown in media with alkanes as the only carbon source. This indicates that these microorganisms have enzymes that can break down alkanes into fatty acids. Researchers have also found that when cerulenin (a fatty acid synthase inhibitor) is added to the medium, the yeast stops using glucose for sophorolipid production. This suggests that the synthesis of sophorolipids involves the creation of new fatty acids within the yeast cells [63].

Sophorose, the hydrophilic part of sophorolipids, is made f rom two UDP-glucoses in a two-step process similar to glycolysis and gluconeogenesis. When combined with the hydroxylated fatty acid side chain, it creates non-acetylated acid sophorolipids. Both water-loving and water-repelling substances can be turned into glucose-6-phosphate through glycolysis and gluconeogenesis. Isocitrate (CIT), a glycolysis intermediate, is transformed into acetyl coenzyme by ATP-citrate lyase (Acl) for the acetylation of sophorolipids. Glucose-1-phosphate is then made from glucose-6-phosphate with the help of phosphoglucose mutase, which reacts with UTP to form UDP-glucose with the help of UDP-glucose pyrophosphorylase. This means that the structure of the sophorolipids hydrophilic part stays the same, even if the sugar sources change. Sophorolipid-producing bacteria can still grow and make sophorolipids even when only given water-repelling carbon sources.

**Biosynthesis of mannosylerythritol lipids.** MELs consist of both hydrophilic and hydrophobic groups. The hydrophilic part is 4-O-β-D-mannopyranosyl erythritol, while the hydrophobic component is a fatty acid chain [65]. Depending on whether acetyl groups are present and where they are located, MELs are divided into four categories: MEL-A, MEL-B, MEL-C, and MEL-D. MEL-A is diacetylated at the C-6 and C-4 positions, MEL-B is monoacetylated at the C-6 position, MEL-C at the C-4 position, and MEL-D is fully deacetylated [66].

The biosynthesis of MELs involves microorganisms utilizing and synthesizing fatty acids. Ther e are three main biosynthetic pathways: (I) de novo synthesis followed by β-oxidation, (II) chain elongation pathway, and (III) intact incorporation pathway [67]. Kitamoto *et al*. [68] investigated the metabolic pathway of MELs biosynthesis by *Candida antarctica* T-34 (*C. antarctica* T-34). They discovered that cerulenin, a potent inhibitor of pathway I, did not significantly affect MELs biosynthesis, indicating that pathway I has a limited role in this process. However, further investigation into pathways II and III revealed that fatty acid formation in MELs was not impacted by cerulenin, indicating that pathways I, II, and III have minimal involvement in fatty acid in fatty acid biosynthesis within MELs. Moreover, Kitamoto *et al*. [67] delved into the impact of the β-oxidation inhibitor 2-bromooctanoic acid (BA) on the synthesis of MELs. Their results showcased a marked inhibitory effect of BA on both the growth of the strain and the production of MELs, with the degree of inhibition increasing in tandem with the concentration of BA. This suggests the presence of two distinct β-oxidation systems in *C. antarctica* T-34: one primarily focused on providing energy and producing acetyl-CoA, which remains unaffected by BA, and another responsible for generating medium-chain fatty acids (C7-C12) crucial for MELs synthesis. Their proposal for a MELs synthesis pathway revolves around the chain-shortening process, as depicted in Fig. 2C: the fatty acid substrate is converted into acyl-CoA, with a portion being directed towards the chain-shortening pathway. The remaining portion is subjected to complete β-oxidation to yield acetyl-CoA, which is further utilized by pathways I and II. In the end, medium-chain fatty acids are created through the chain-shortening process and then combined with mannose and erythritol, which are produced through pathway I, to produce MELs.

Hewald *et al*. [69] explored the roles of two key genes, *emt1*, and *cyp1*, in the regulation of MELs synthesis in *Ustilago maydis*. These genes are crucial components of the extracellular glycolipid biosynthesis pathway in fungi. The *emt1* gene plays a vital role in MELs synthesis by encoding a protein that functions similarly to protoribosyltransferase, facilitating guanosine-5'-diphosphate-mannose (GDP-mannose) transfer essential for MELs biosynthesis and the production of macrolide antibiotics. On the other hand, *cyp1* is linked to plant fatty acid hydroxylase, encoding a cytochrome P450 monooxygenase (CytP450) that plays a role in directing the synthesis of 15,16-dihydroxyhexadecanoic acid [70]. Deletion of *cyp1* leads to the strain being unable to synthesize fenamic acid. In addition, Hewald *et al*. [71] examined glycosyltransferase Emt1, which is crucial for MELs production and is upregulated in response to nitrogen deficiency. The Emt1 gene cluster consists of five open reading frames, including Mac1, Mac2, and Mat1, newly discovered proteins with specific sequences typical of acyltransferases and acetyltransferases. Hewald *et al*. [71] discovered that genes *mac1*, *mac2*, and *mat1* are crucial for MEL synthesis through mutation analysis and mass spectrometry. Specifically, *mac1* and *mac2* control MEL acylation, while *mat1* influences MEL acetylation. Following this, Hewald *et al*. [71] conducted an experiment where they overexpressed the gene *mat1*. Their findings revealed that *mat1* acts as an acetyl-CoA-dependent acetyltransferase, with the ability to acetylate MELs at the C-4 and C-6 positions. Therefore, a proposed pathway for MELs synthesis in *U. maydis* [71] involves the initial condensation of mannose and erythritol catalyzed by glycosyltransferase Emt1. Following this, Mac1 and Mac2 assist in transferring medium and short-chain fatty acids to the C-2 and C-3 positions. Finally, Mat1 catalyzes the acetylation of the C-4 and C-6 positions of deacetylated MELs, resulting in various configurations of MELs.

**Biosynthesis of trehalolipids.** Trehalolipids, which are a type of glycolipid biosurfactants, have many benefits including low toxicity [72], excellent stability [73], resistance to acid and salt [74], biodegradability [75], eco-friendliness [76], and antibacterial properties [77]. They are mainly produced by Gram-positive bacteria and actinomycetes with a high GC content, such as *Mycobacteria*, *Nocardia*, *Rhodococcus*, and *Corynebacteria* [78-81]. Trehalolipids, like other biosurfactants, have a basic structure consisting of both hydrophilic and hydrophobic components. The hydrophilic part is made up of a non-reducing disaccharide, connecting two glucose units through α, α-1,1-glycosidic bonds. On the other hand, the hydrophobic portion is more diverse [82], including fatty acids of different chain lengths and hydroxylated branched fatty acids, forming monoesters, diesters, and tetraesters respectively [83-85]. Trehalolipids obtained from various microbial sources can vary in size, structure, carbon atom number, and level of unsaturation. Among these, trehalose-6,6'-dimycolipid (TDM), trehalose-6-monomycolipid (TMM), and succinyl trehalose lipids (STL) are the most extensively studied [86].

Kretschmer and Wagner proposed the biosynthetic pathway of trehalose-6,6'-dimycolic acid ester (TDM), as shown in Fig. 2D. The process starts with converting n-alkanes into n-alkanoic acids, which then undergo β-oxidation to produce acetyl-CoA. Acetyl-CoA is then used in gluconeogenesis to form glucose 6-phosphate. 6-phosphate trehalose synthase (TPS) transfers UDP-glucose to glucose 6-phosphate to create 6-phosphate trehalose, which forms the hydrophilic part of trehalolipids. Meanwhile, n-alkanoic acids combine to form 3-oxo-2-dodecyl-docosanoic acid, which is then converted into trehalose-6-phosphate monomycolate by cytoplasmic acyltransferase activity. The phosphate group is removed by phosphatase to produce trehalose-6-mycolate (TMM). TMM is transported outside the cell through an ABC transporter, and with the assistance of extracellular mycolyltransferase (Ag85/Fbp/PS1), trehalose-6,6'-dimycolate is synthesized [86-88].

### Biosynthesis of Lipopeptides

Lipopeptide biosurfactants, which consist of hydrophilic peptide chains and lipophilic fatty acid chains, are predominantly synthesized by microorganisms like *Bacillus* (such as *Bacillus subtilis*, *Bacillus licheniformis* (*B. licheniformis*), and *Bacillus amyloliquefaciens*), *Pseudomonas*, *Streptomyces*, *Aspergillus*, *Serratia*, and *Actinomycetes* [89]. Lipopeptides are secondary metabolites produced by *Bacillus* using a complex multi-enzyme system called non-ribosomal peptide synthetase (NRPS) [90]. They can be categorized into two main groups based on their chemical structure: cyclic lipopeptides [91] and linear lipopeptides [92]. Cyclic lipopeptides have a circular molecular structure formed by different methods of ring closure between fatty acid and peptide rings, while linear lipopeptides are made up of a linear chain of fatty acids and amino acids without any cyclic structures [10]. There are currently about 90 lipopeptides known to belong to 26 homologues, with the majority being cyclic lipopeptides (86 compounds in 24 homologues) and only four compounds classified as linear lipopeptides [93]. Among these homologues, surfactin [94], lichenysin [95], iturin [96] and fengycin [97] have been extensively studied.

**Biosynthesis of surfactin.** Surfactin, a cyclic lipopeptide biosurfactant, is considered one of the most powerful biosurfactants due to its composition of a seven-membered peptide ring containing four types of amino acid residues and their isomers (L-Glu → L-Leu → D-Leu → L-Val → L-Asp → D-Leu → L-Leu) linked to a 13-16 carbon β-hydroxy fatty acid chain via a lactone bond [98]. The hydrophilicity of surfactin mainly comes from its interactions involving carboxyl, peptide bond, and internal lipid bond. Particularly, the first glutamic acid (Glu) and the fifth aspartic acid (Asp) are dicarboxylic amino acids, known as acidic amino acids. Glu also contains a γ-carboxyl group, which significantly contributes to the hydrophilic nature of surfactin. The side chains of other amino acid residues, such as valine and leucine, are made up of saturated aliphatic hydrocarbon chains, which further enhance hydrophobicity alongside the β-hydroxy fatty acid chain. Because of differences in fatty acid chain length and amino acid composition and positioning, surfactin displays a range of homologues and isomers, such as C13, C14, C15, and C16 variants[99]. Researchers have used the mutant strain BS-37 of *B. subtilis* to produce surfactin homologues, mainly consisting of C15 surfactin (comprising elements of both C13 and C14), with the composition ratio of the surfactant mixture changing depending on the culture media. A higher proportion of C15 surfactin in the product is associated with an increase in oil recovery efficiency [100].

The surfactin synthase gene open reading frame contains *srfAA*, *srfAB*, *srfAC*, and *srfAD*, coverin g a total length of 27 Kbp [101, 102]. Each NRPS module is responsible for the recognition and condensation of specific amino acids [103-105], and consists of several domains: A domain (adenosine), T/PCP domain (thiolation), C domain (condensation), and TE domain (thioesterification) [105-107]. The *srfAA* and *srfAB* gene clusters have three modules each, while *srfAC* has one module. The A domain in modules A1, A3, B2, and B3 shows high specificity, recognizing L-Glu, L-Leu, L-Asp, and L-Leu, respectively. Modules A3 and B3 also have an epimerization domain (E domain) that converts L-Leu to D-Leu [108]. The cyclization and release of surfactin are facilitated by the type II thioesterase gene, *srfAD*, located downstream of *srfAC* [109]. Additionally, the *srfA* operon includes a phosphopantetheinyl transferase (PPTases) gene, *sfp*, crucial for activating surfactin synthase and facilitating surfactin synthesis [110, 111].

**Fig. 3:**
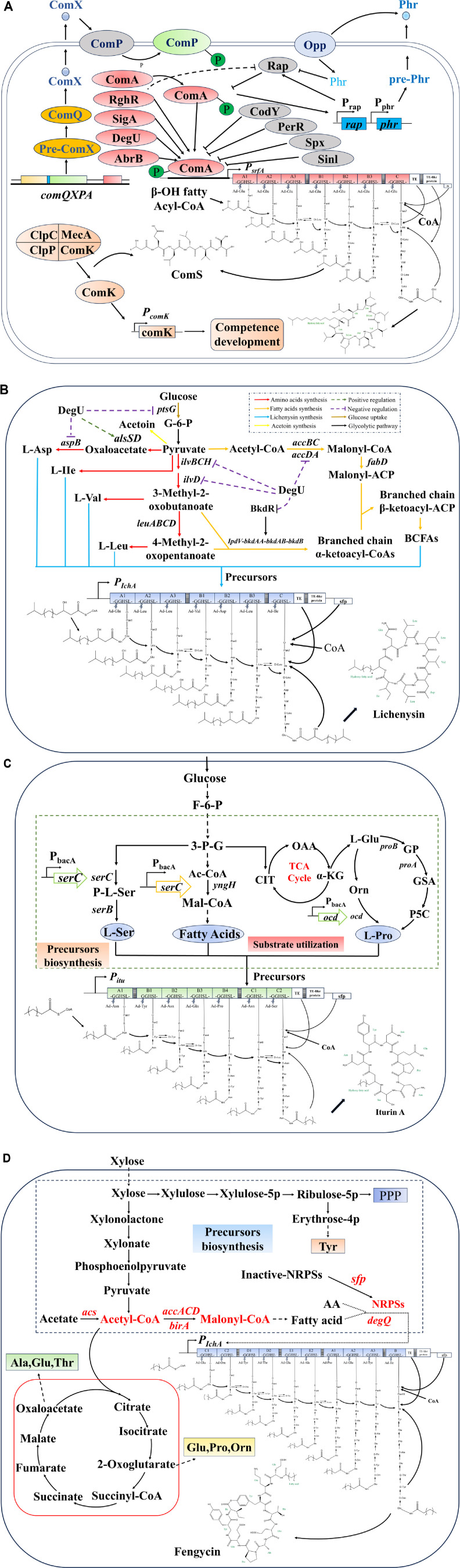
The biosynthesis pathway of surfactin (A) lichenysin (B) iturin A (C) and fengycin (D).

Surfactin synthesis a nd regulation constitute a complex cascade of reactions mediated by quorum sensing [102, 112, 113]. This process is intricately linked to natural competence, spore formation and cell motility in *Bacillus* [114, 115], as shown in Fig. 3A. As a signaling molecule indicative of cell density, the *Bacillus* pheromone ComX increases in concentration with cell growth. Upon reaching a threshold concentration, ComX binds to the membrane protein histidine kinase ComP, triggering autophosphorylation. Phosphorylated ComP subsequently transfers the phosphate group to the regulatory protein ComA. Phosphorylated ComA then binds to the specific promoter region of the surfactin synthase gene *srfA*, activating RNA polymerase and initiating transcription [116-118]. The transcriptional expression of the *comS* gene, embedded within the *srfAB* gene cluster, marks the onset of competence in *Bacillus* cells [119]. Thus, surfactin synthesis is intricately tied to competence formation. In addition to ComX, *B. subtilis* produces competence stimulating factor (CSF), a peptide containing five amino acids (ERGMT), which regulates surfactin synthesis in tandem with increasing cell density [120-122]. CSF is encoded by the *phr* gene, with an upstream rap gene encoding aspartic acid phosphatase. The Phr precursor peptide is exported from the cell via an unknown mechanism and subsequently imported into the cell through oligopeptide permease Opp, where it binds to Rap protein and inhibits its phosphatase activity [123]. In *B. subtilis*, there are 11 Rap proteins, with seven of them (RapA, RapC, RapE, RapF, RapG, RapI, RapK) specifically antagonized by corresponding Phr peptides [124]. Building upon this framework, several studies have effectively enhanced surfactin production by engineering strains that overexpress ComX and PhrC [125]. The expression of the *comS* gene, which is essential for *Bacillus* to become competent, is closely connected to the expression of the surfactin synthase gene. This connection is crucial for *Bacillus* to maintain its population advantage in natural environments [99]. Surfactin acts as an antibiotic, helping *Bacillus* eliminate competing bacteria and allowing for the release of nutrients and genetic material [126]. Competence formation allows *Bacillus* to absorb genetic material released by other microorganisms, improving its capabilities and strengthening its competitive edge for survival [127].

In *Bacillus*, the presence of ComX and CSF pheromones triggers a sustained increase in intracellular phosphorylated ComA levels. This ultimately leads to the formation of ComA tetramers that bind to the *srfA* gene promoter region (ComA Box, TGCGG-N4-CCGCA), interacting with SigA to activate the transcription of the *srfA* operon [113]. Rap proteins and their corresponding Phr peptides indirectly control the production of surfactin by influencing the levels of ComA phosphorylation [128]. New studies have shown that the transcription factor RghR, which plays a role in regulating spore formation, works to block Rap protein activity, ultimately leading to an increase in surfactin synthesis [129]. The global regulator DegU also positively influences surfactin synthesis [113, 130], as evidenced by significant reductions in *srfA* transcription upon *degU* gene knockout. Conversely, CodY acts as a negative regulator of *srfA* transcription [131], with its knockout substantially enhancing surfactin production, confirming its repressive role [132]. The regulation of *srfA* transcription in *Bacillus*, especially in relation to surfactin synthesis, is likely influenced by additional regulators such as AbrB, Spx, PerR, PhoP, and SinI. However, the specific roles of these regulators remain unclear and are mainly inferred from structural predictions or omics data. As a result, our understanding of the key regulatory factors and mechanisms involved in efficient surfactin synthesis is still developing.

**Biosynthesis of lichenysin.** Lichenysin is a type of lipopeptide biosurfactant produced by *B. licheniformis*. It has a structure similar to other surfactants, with both polar hydrophilic and non-polar hydrophobic groups made up of aliphatic hydrocarbon chains. Natural lichenysin is a mixture of different homologues with varying lengths of fatty acid side chains, resulting in a molecular weight ranging from 993 Da to 1049 Da [133]. It consists of seven amino acids linked in a peptide chain, forming a three-dimensional saddle-shaped structure [134]. The typical sequence of amino acids is L-Gln → D-Leu → L-Leu → L-Val → L-Asp → D-Leu → L-Ile [133]. The seventh amino acid in the chain forms a ring with the β-hydroxy fatty acid through a lactone bond, with the fatty acid chain length ranging from C12 to C17 [133]. In terms of structure, lichenysin is very similar to surfactin, with the main difference being the first amino acid in their peptide chains.: L-Gln for lichenysin and L-Glu for surfactin [135], which significantly impacts their respective properties. Grangemard *et al*. [136] demonstrated that the critical micelle concentration (CMC) of lichenysin G (25 μmol/l) in a 5 mmol/l Tris buffer was found to be approximately 10 times lower than that of surfactin (220 μmol/l), with lichenysin G (0.39 nm^2^) occupying about half the molecular area compared to surfactin (1.01 nm^2^). Nonribosomal peptide synthetase (NRPS) plays a key role in the synthesis pathways of various antibiotics, including lichenysin. The lichenysin synthase gene operon, *lchA*, consists mainly of four open reading frames (ORFs): *lchAA*, *lchAB*, *lchAC*, and *lchA-TE*. These ORFs encode the subunits of lichenysin synthase: LchAA, LchAB, LchAC, and the thioesterase LchA-TE, respectively. Each ORF is responsible for the specific recognition of amino acids and aids in the orderly synthesis of lichenysin [133].

Hu *et al*. [137]. proposed the biosynthetic pathway of lichenysin, as shown in Fig. 3B. Intermediate products of glycolysis are converted into various amino acids and branched-chain fatty acids essential for lichenysin biosynthesis. Pyruvate is transformed into aspartic acid (L-Asp) and isoleucine (L-Ile), while 3-methyl-2-oxobutanoate is converted into valine (L-Val) and 4-methyl-2-oxopentanoate into leucine (L-Leu). These synthesized amino acids serve as substrates for the subsequent lichenysin synthesis. Pyruvic acid and 4-methyl-2-oxopentanoate are converted into branched-chain fatty acids (BCFAs) under the regulation of genes *accBC*, *accDA*, and *fabD*. The synthesized amino acids and BCFAs collectively form the precursors for lichenysin, which is then synthesized by NRPS.

**Biosynthesis of iturin.** Iturin, a cyclic lipopeptide biosurfactant, is defined by its molecular structure consisting of seven amino acid residues and a β-amino fatty acid chain with 14-17 carbons. More specifically, the initial amino group Asn is combined with the carboxyl group of the β-amino fatty acid, while the seventh carboxyl group Ser is combined with the β-amino group on the fatty acid side chain, resulting in a cyclic formation [138]. The amino acid sequence of the heptapeptide is (L-) Asn- (D-) Tyr- (D-) Asn- (L-) Gln- (L-) Pro (D-) Asn- (L-) Ser. Variants such as iturin A, C, D, and E exhibit differing fatty acid chains of various carbon lengths [139]. If the positions of Asn at the sixth and Ser at the seventh are exchanged, the resulting homolog is known as mycosubtilin [140]. Additionally, the iturin family encompasses Bacillomycin D and Bacillopeptin, featuring variations in amino acids at the third, fourth and fifth positions [141, 142], as shown in Table 1.

The iturin A synthetase operon consists of four open reading frames (ORFs): *ituD*, *ituA*, *ituB*, and *ituC*, which are about 38 kb in length [143]. Transcription is initiated by the promoter P_itu_. The *ituD* gene codes for a malonyl-CoA transacylase involved in fatty acid synthesis. Disrupting this gene leads to a decrease in iturin A production [144]. The *ituA* gene encodes a 449 kDa protein with three functional modules similar to fatty acid synthase, amino acid transferase, and peptide synthase [145]. The third and fourth genes, *ituB* and *ituC*, encode peptide synthetases weighing 609 kDa and 297 kDa respectively [144]. These enzymes play a key role in assembling amino acid modules necessary for the synthesis of iturin A [144].

The proposed biosynthetic pathway of iturin was outlined by She *et al*. [145], as shown in Fig. 3C: Intermediate products from glycolysis are transformed into iturin precursors through a series of enzymatic reactions. Specifically, 3-phosphoglycerate is converted to serine (L-Ser) by the *serA*, *serC*, and *serB* genes [146]. Acetyl CoA is converted to malonyl CoA by the *yngH* gene, which then forms branched-chain fatty acids [147]. At the same time, acetyl CoA enters the TCA cycle to produce proline (L-Pro). These amino acids and fatty acids are used as the building blocks for iturin synthesis, which is facilitated by the iturin biosynthetic gene cluster [145].

**Biosynthesis of fengycin.** Fengycin is an antimicrobial cyclic lipopeptide, consisting of 10 amino acids and 14-18 carbon atoms of β-hydroxy fatty acids [150, 151]. Unlike the typical peptide synthesis method, the biosynthesis of fengycin in *B. amyloliquefaciens* employs a five-enzyme subunit system to carry out its production through enzymatic processes [152]. The enzyme complex, consisting of FenA, FenB, FenC, FenD, and FenE subunits, utilizes a multi-carrier thiolation template mechanism within the NRPS family to synthesize fengycin [153]. Due to its nature as a secondary metabolite, fengycin is not synthesized by ribosomes in the same way as an antimicrobial peptide. Its production requires a complex intracellular enzymatic synthesis process that is influenced by various factors.

The fenA-E gene cluster, which encodes fengycin synthase, forms a *fen* operon that spans around 37 kb [153]. Each subunit typically contains 1 to 3 amino acid activation modules that are specific to particular amino acids [154]. The first gene in the fengycin synthase pathway is *fenC*, which contains an enzyme consisting of two activation modules: FenC1 activates L-glutamic acid (L-Glu), and FenC2 activates L-ornithine (L-Orn) [152]. The second gene, *fenD*, encodes an enzyme with two activation modules: FenD1 for L-tyrosine (L-Tyr) and FenD2 for L-threonine (L-Thr) [155]. The third gene is *fenE*, encoding an enzyme, which carries two activation modules: FenE1 for L-glutamic acid (L-Glu) and FenE2 for L-alanine (L-Ala) and L-valine (L-Val) [156]. The fourth gene *fenA* is composed of three activation modules: FenA1 for L-proline (L-Pro), FenA2 for L-glutamic acid (L-Glu), and FenA3 for D-tyrosine (D-Tyr), allowing for their incorporation into the peptide chain. The final one, *fenB*, is responsible for activating L-isoleucine (L-IIe) [157]. After the last amino acid is added to the peptide chain, an intramolecular nucleophilic attack occurs, resulting in the formation of the cyclization product. FenB contains a thioesterase domain at its C-terminus, which aids in releasing the peptide chain and ending the reaction [157].

Tan *et al*. [158] proposed the biosynthetic pathway of Fengycin, as shown in Fig. 3D: Acetyl-CoA produced from glycolysis enters the citric acid cycle. 2-ketoglutaric acid is converted into L-glutamic acid (L-Glu), L-proline (L-Pro), and L-ornithine (L-Orn). Oxaloacetic acid is transformed into L-alanine (L-Ala), L-glutamic acid (L-Glu), and L-threonine (L-Thr). Erythrose 4-phosphate, generated through the pentose phosphate pathway, is converted into L-tyrosine (L-Tyr). These amino acids are then used in the synthesis of Fengycin peptides. Acetyl-CoA is converted to malonyl-CoA by the enzymes accACD and birA, which is then further transformed into branched-chain fatty acids. Fengycin is produced from amino acids and branched-chain fatty acids using NRPS and the *degQ* gene.

### Biosynthesis of Phospholipids

Phospholipids are a type of lipid that have both hydrophilic and hydrophobic properties. They consist of a nitrogen- or phosphorus-containing head group and a long-chain fatty acid tail. The backbone of phospholipids is typically glycerol, with two acyl groups attached to the C-1 and C-2 positions, and a phosphorylated hydroxyl group at the C-3 position. The phosphate group connects the glycerol backbone to the polar head group, which can be choline, ethanolamine, inositol, serine, or glycerol. Different combinations of head groups result in different subclasses of phospholipids, such as phosphatidylcholine (PC), phosphatidylethanolamine (PE), phosphatidylinositol (PI), phosphatidylserine (PS), and phosphatidylglycerol (PG). Phospholipids are known for their emulsification properties and are commonly used as emulsifiers in food, cosmetics, and pharmaceutical products [159].

**Fig. 4:**
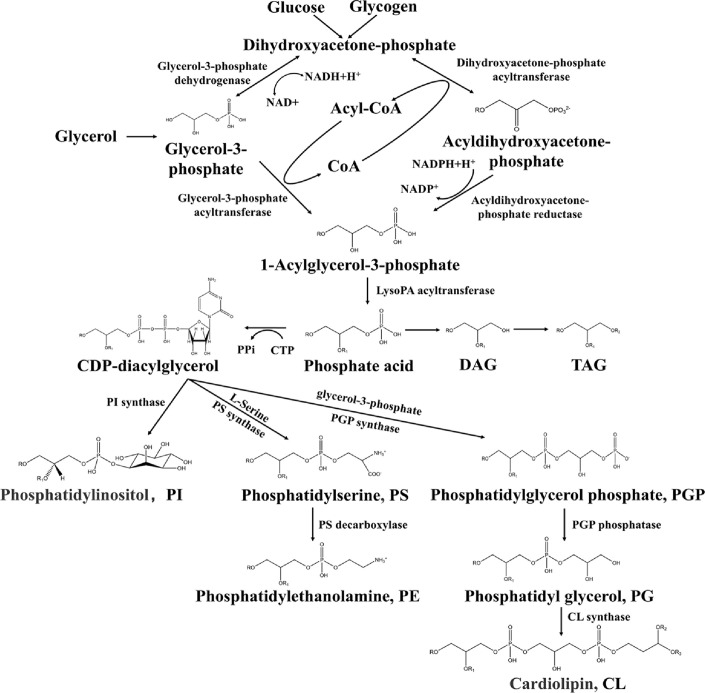
The biosynthesis pathway of phospholipids.

The biosynthetic pathway of phospholipids begins with the conversion of glucose and glycogen into dihydroxyacetone phosphate (DHAP), as shown in Fig. 4. DHAP is then converted into 3-phosphoglycerol (3-PG) or acyldihydroxyacetone-phosphate (acyl-DHAP) by 3-PG dehydrogenase (3-PGDH) or DHAP acyltransferase (DGAP-AT), respectively. These intermediates are further processed into 3-phospho-1-acylglycerol (3-P-1-AG) via 3-PG acyltransferase (3-PGAT) or DHAP reductase. Finally, 3-P-1-AG is converted to phosphatidic acid (PA) by 3-P-1-AG acyltransferase (3-P-1-AG-AT). PA serves as the precursor for major phospholipids - PC, PE, PI, PS, and PG - depending on the polar group attached via the phosphate group [159].

### Biosynthesis of Polymeric Biosurfactants

Polymeric biosurfactants are usually made up of high-molecular-weight biopolymers that are recognized for their rigidity, heightened viscosity, and resistance to shear. Emulsans and lipopolysaccharides produced by *Acinetobacter calcoaceticus* and *Candida lipolytica* are the most widely researched macromolecular biosurfactants in this category [160].

**Biosynthesis of lipopolysaccharide.** Lipopolysaccharide (LPS) is a water-soluble emulsifier made by *C. lipolytica*, comprising lipid A, core polysaccharide, and the O-antigen (OAg) repeat sequence [161, 162]. LPS acts as a vital permeability barrier that protects Gram-negative bacteria from their surroundings [163]. The OAg, prominently featured on the LPS surface, is crucial in increasing pathogenicity. It protects invading bacteria from host bactericidal responses [164, 165], regulates macrophage recognition[166], facilitates epithelial cell invasion [167, 168], and supports intracellular survival [169].

**Fig. 5:**
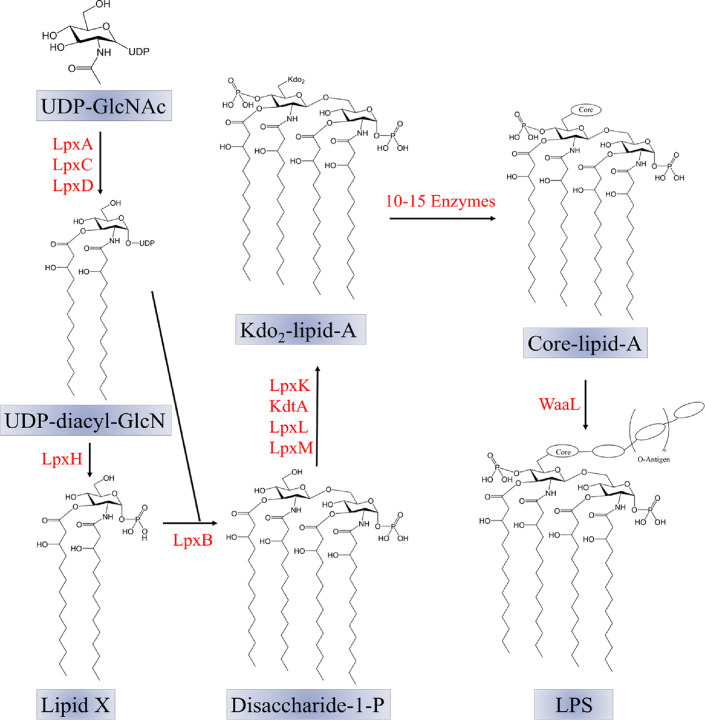
The biosynthetic pathway of lipopolysaccharide.

The biosynthesis of LPS starts with UDP-N-acetylglucosamine (UDP-GlcNAc) as the initial molecule, as shown in Fig. 5. A series of enzymes then convert UDP-GlcNAc into disaccharide-1-P, Kdo_2_-lipid A, core-lipid A, and finally, LPS. The process of creating Kdo_2_-lipid A requires the action of nine enzymes. Soluble enzymes like UDP-N-acetylglucosamine acyltransferase (LpxA), UDP-3-O-[(R)-3-hydroxymyristoyl]-N-acetylglucosamine deacetylase (LpxC), and UDP-3-O-(R-3-hydroxyacyl)GlcN N-acyltransferase (LpxD) facilitate the addition of two 3-OH fatty acid chains to the second and third positions of UDP-GlcNAc, forming UDP-diacyl GlcN [170-172]. Subsequently, LpxH hydrolyzes UDP-diacyl GlcN to produce lipid X [173, 174]. Lipid X and its precursor, UDP-diacyl-GlcN, are condensed to form 1-phosphate-disaccharide under the catalysis of LpxB [175, 176]. This pathway also involves enzymes like tetraacyldisaccharide-1-phosphate 4'-kinase (LpxK), 3‐Deoxy‐D‐manno‐oct‐2‐ulosonic acid (Kdo) transferases (KdtA), Kdo_2_-lipid IVA lauroyl-ACP acyltransferase (LpxL), and Kdo_2_-lauroyl-lipid IVA myristoyl-ACP acyltransferase (LpxM) to catalyze subsequent reactions. The 4'-position of 1-phosphorylated disaccharide is then phosphorylated to produce lipid IVA with the help of LpxK [177, 178]. Following that, KdtA attaches two 3-deoxy-D-manno-octulosonic acid (Kdo) residues at the 6'-position of lipid IVA to create Kdo_2_-lipid IV A [179]. This intermediate is further modified by LpxL and LpxM to form Kdo_2_-lipid A. The genes essential for core oligosaccharide biosynthesis are organized into three operons: *gmhD*, *waaQ*, and *kdtA* [180]. In *E. coli* K-12, the *gmhD* operon comprises four genes (*gmhD*-*waaF*-*waaC*-*waaL*) necessary for core oligosaccharide biosynthesis [181]. Specifically, *gmhD*, *waaF*, and *waaC* encode proteins involved in Hep biosynthesis and transport, while *waaL* encodes the ligase responsible for linking the O antigen to core lipid A [182]. The *waaQ* operon consists of 7 to 9 genes encoding enzymes responsible for the synthesis and modification of outer core oligosaccharides. The *kdtA* gene within the *kdtA* operon encodes KdtA, which adds two Kdo residues to lipid IVA [179]. O antigens, similar to core oligosaccharides, are produced on the cytoplasmic side of the inner membrane and attached to a membrane-bound carrier by glycosyltransferases using sugar nucleotides as donors [183]. The *rfb* gene cluster in *E. coli* and *Salmonella enteritidis* contains the necessary enzymes for creating sugar nucleotide precursors specific to O antigens, glycosyltransferases, polymerases for assembling O antigens, and proteins for transporting O antigen polymers across the inner membrane [184]. After being synthesized on the cytoplasmic side, both core lipid A and O antigens are moved to the periplasmic side of the inner membrane. The O antigen is then polymerized by Wzy and Wzz and connected to core lipid A through WaaL to create mature LPS [185].

**Biosynthesis of emulsan.** Emulsan biosurfactant is a complex acylated polysaccharide, containing polysaccharides, D-aminogalactose, D-aminogalacturonic acid, and D-dideoxy-diaminohexose in specific ratios. Its amphiphilic properties, stemming from a hydrophilic carbohydrate chain and hydrophobic fatty acid side chains, make emulsan a highly efficient bioemulsifier [186].

**Fig. 6:**
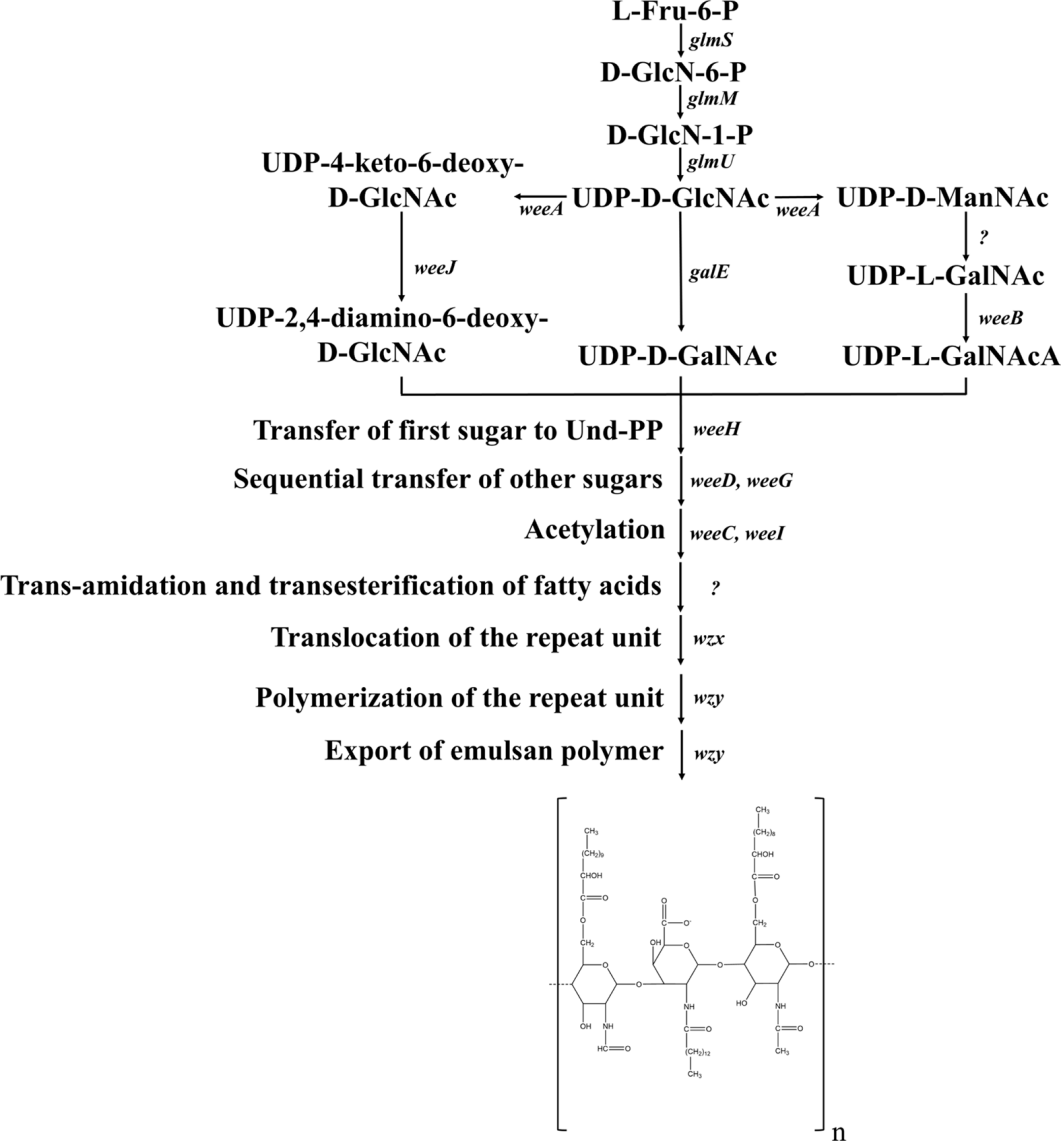
The biosynthesis pathway of emulsan. The question mark indicates that no gene catalyzing the reaction has been found or the function of the gene has not been confirmed.

The biosynthetic pathway of emulsan, as shown in Fig. 6, starts with the conversion of L-6-phosphate fructose to D-UDP-acetylglucosamine through the action of genes *glmS*, *glmM*, and *glmU*. Next, D-UDP-acetylglucosamine is converted to D-UDP-acetylgalactosamine by the gene *galE*. Genes *weeK* and *weeJ* then catalyze the formation of UDP-2,4-diamino-6-deoxy-D-acetylglucosamine, which is further transformed into UDP-L-acetylgalactosaminic acid by *weeA* and *weeB*. These processes involve glycosyl conversion and acetylation of amino acids coordinated by genes *weeH*, *weeD*, *weeG*, *weeC*, and *weel* [187]. Moreover, the genes *wzx*, *wzy*, *wza*, *wzb*, and *wzc* play a crucial role in regulating the transamidation and transesterification of fatty acids, as well as the transfer and polymerization of repeat units, and the secretion of lipid emulsion polymers [187].

## Metal Ions Regulate the Biosynthesis of Biosurfactants

The production of biosurfactants through fermentation is influenced by various factors, such as medium composition [188], temperature [189], pH [190], feeding schedule [191], and others. Among these factors, medium composition is considered the most important factor affecting biosurfactant fermentation production. Metal ions are crucial components of the medium composition as they provide essential nutrients for biological activities and regulate strain metabolism. However, current research mainly focuses on the overall effects of metal ions on biosurfactant production, with limited exploration into the specific molecular pathways involved in biosurfactant synthesis. Therefore, this section aims to investigate how metal ions impact the biosurfactant synthesis pathways at a molecular level.

### Metal Ions Regulate the Biosynthesis of Glycolipids

Metal ions usually have a direct impact on microbial growth or can activate genes related to glycolipid synthesis, ultimately affecting glycolipid production, as shown in Table 2. Déziel *et al*. [192] used ammonium salt as the nitrogen source in their experiment and added extra iron ions to the medium. They found that nitrate nitrogen increased the expression of rhamnosyl transferases RhlA and RhlB in *P. aeruginosa*, while ammonium nitrogen suppressed these genes. Higher levels of iron in the medium resulted in decreased RhlA expression, suggesting that nitrate nitrogen promoted rhamnolipid production, whereas ammonium nitrogen and excess iron inhibited glycolipid production. Additionally, limitations in iron ion concentrations had a significant impact on the regulation of quorum sensing system-related genes *rhlI* and *rhlR* in *P. aeruginosa* [193]. In another study [194], the levels of *rhlB*/*rhlC* expression and the production ratio of single rhamnolipid (RL1) to double rhamnolipid (RL2) in *P. aeruginosa* IGB83 were examined at Cd^2+^ concentrations of 0.45 mM and 0.89 mM. The findings showed that Cd^2+^ triggered the expression of RhlB and led to an increase in the RL2/RL1 production ratio. The timing of adding nutrients and metal ions also influenced rhamnolipid production. higher yields were obtained when (NH_4_)_2_SO_4_ and trace metals were added gradually rather than all at once [195]. Chen *et al*. [196] investigated the effects of different metal ions (Mg^2+^, Zn^2+^, Fe^2+^, Cu^2+^, Fe^3+^, Mn^2+^, Ni^2+^) on cell growth and sophorolipid production in *Wickerhamiella domercqiae* Y2A CGMCC 3798 were studied. It was found that Cu^2+^ had the most significant impact, leading to approximately 140.82 g/l production. Furthermore, Mg^2+^ supplementation resulted in a higher proportion of lactone-type sophorolipids, while Fe^2+^ was the optimal ions for acidic sophorolipids production. Chen *et al*. [196] used SDS-PAGE and A-SLs transformation methods to suggest that Fe^2+^ and Mg^2+^ could either enhance or hinder the enzymes involved in the SLs metabolic pathway of *W. domercqiae*, leading to alterations in SLs composition and production. The enzyme is possibly a lactone esterase or another type of regulatory protein [62]. Additionally, research has demonstrated that metal ions like Mg^2+^, Cu^2+^, Fe^2+^, Ca^2+^, and Mn^2+^ play a role in controlling the production and structure of MELs produced by *Pseudozyma aphidis*, *Ceriporia lacerate*, and *Moesziomyces aphidis* [197, 198]. Mg^2+^ and Mn^2+^ can both significantly enhance the yield of MELs synthesis. Despite this, there is a lack of studies on how metal ions influence the biosynthesis process of MELs, underscoring the requirement for more research.

### Metal Ions Regulate the Biosynthesis of Lipopeptides

The control of metal ions on lipopeptide biosurfactants typically entails the modulation of proteins associated with carbon and nitrogen metabolism or the direct stimulation of genes responsible for lipopeptide synthesis, thus impacting their production, as shown in Table 3. Cooper *et al*. [199] observed that the addition of Fe^2+^ and Mn^2+^ to *B. subtilis* medium resulted in an increase in surfactin production. However, the addition of Mn^2+^ only enhanced the production of surfactin without impacting the strain's biomass [200]. Conversely, the excessive addition of Fe^2+^ not only boosted surfactin synthesis but also stimulated strain growth, which was unexpected. As a result, it is suggested that the regulation mechanism for these two metal ions is not the same. *B. subtilis* might not have an Fe^2+^ transport system or may produce chelating compounds that prevent Fe^2+^ utilization [201]. The synthesis of surfactin results in the formation of a chelate with Fe^2+^ that is then taken up by cells to stimulate growth. Mn^2+^ is an essential trace metal for the growth of certain *Bacillus* and fungi, serving as a cofactor for numerous nitrogen metabolism enzymes [202]. The addition of this component greatly changes the way strain nitrogen metabolism works, improving the utilization of NH_4_^+^. and NO_3_^-^. When Mn^2+^ concentrations are increased, the utilization of NO_3_^-^ is enhanced, leading to a shift in which it becomes the main nitrogen source instead of NH_4_^+^. This change results in an increase in surfactin production [28]. Mg^2+^ and K^+^ also influence strain growth and surfactant formation [203]. The peptide carrier protein domain of *B. subtilis* ATCC 21332 Sfp protein, activating surfactin synthase, accommodates Mg^2+^ as a cofactor [111], highlighting the importance of Mg^2+^ in surfactin synthesis. K^+^ has the ability to activate the surfactin synthesis gene *srfA*, promoting surfactin secretion, which could increase overall production and enhance *B. subtilis* motility [204, 205]. Adding Ca^2+^ to strain FJAT-4 has an impact on its growth and the synthesis of lipopeptides like surfactin and fengycin. Even though elevated Ca^2+^ levels do not hinder the growth of FJAT-4, they do suppress the expression of genes related to lipopeptide synthesis, ultimately reducing the production of lipopeptides. Proposed regulatory pathways, including restriction endonuclease regulation system ResE/ResD, phosphorylase regulation system PhoR/PhoP, differentially expressed genes regulation system DegS/DegU elucidate how Ca^2+^ reduces lipopeptide synthesis [206]. Lin *et al*. [207] adjusted Fe^2+^ concentrations and pH to regulate iturin production. At pH 6.64 and 0.2 mM Fe^2+^, they achieved the highest yield of iturin A at 121.28 mg/l, surpassing previous reports for *B. amyloliquefaciens*.

### Metal Ions Regulate the Biosynthesis of Phospholipids

Metal ion regulation of phospholipid biosurfactants primarily involves direct induction of genes related to phospholipid synthesis, impacting phospholipid yield or structure, as shown in Table 4. Zn^2+^ complexes with the phospholipid head group, thereby reducing its hydrophilicity [208]. Exposure to Cadmium (Cd^2+^) significantly increased phosphatidylcholine levels in mouse serum and hepatocytes. Metabolomics analysis revealed up-regulation of lysophosphatidylcholine acyltransferase 3 (LPCAT3), phosphate cytidylyltransferase 1 choline alpha (PCYT1A), and phosphatidylethanolamine N-methyltransferase (PEMT), key enzymes in phosphatidylcholine biosynthesis [209]. Ca^2+^ inhibits the activity of lysophosphatidylcholine acyltransferase 1 (LPCAT1) in the phosphatidylcholine synthesis pathway, and this regulation depends on a specific EF-hand Ca^2+^ binding motif (EFh-1) located at the end of the protein [210]. The loop within EFh-1, composed of Asp-392 and Glu-403 residues, is crucial for this process. When these residues are replaced with alanine (D392 and E403), the enzyme becomes unresponsive to Ca^2+^, indicating that Ca^2+^ binding to EFh-1 negatively affects the activity of the amino terminal domain of acyl-CoA LPCAT1. This regulation ultimately impacts the synthesis of phosphatidylcholine in cells [210]. Within *P. aeruginosa*, phosphorylcholine phosphatase (PchP) plays a key role in converting phosphocholine or sphingomyelin into choline and inorganic phosphate. The activity of PchP is strongly influenced by the concentrations of Mg^2+^ and Zn^2+^, with a marked enhancement observed at pH levels below 6.0. Conversely, Zn^2+^ hinders PchP activity in neutral to alkaline conditions, thereby impacting the generation of choline and phosphate [211]. Silver (Ag^+^), Copper (Cu^2+^), Mercury (Hg^2+^), and Lead (Pb^2+^) form thiols with cysteine (Cys), thereby inhibiting lyso-PC acyltransferase activity [212]. Taniguchi *et al*. [213] studied the impact of Ca^2+^ on the activity of ethanolamine phosphate and choline phosphate transferase in platelet cells, which depend on Mn^2+^, Co^2+^, or Mg^2+^ as cofactors. The findings indicated that Ca^2+^ could directly bind to the metal cofactor binding sites of ethanolamine phosphate transferase and choline phosphate transferase in the presence of optimal Mg^2+^ concentration, leading to enzyme inhibition and decreased production of ethanolamine phosphate and choline phosphate.

### Metal Ions Regulate the Biosynthesis of Polymeric Biosurfactants

The regulation of polymeric biosurfactants is primarily influenced by metal ions, which modulate cellular immune defense systems and, consequently, impact surfactant synthesis, as shown in Table 5. Silver nanoparticles (AgNPs) demonstrate strong antibacterial properties by inducing bacterial resistance to the surface charge of the nanoparticles. This resistance mechanism, referred to as the envelope stress response (ESR), involves the modification of lipopolysaccharide (LPS) and its key component, lipid A. Essentially, AgNPs indirectly control LPS synthesis by exploiting bacterial immune defense mechanisms [214]. Lu *et al*. [215] analyzed the transcriptome of *E. coli* K-12 LE392 after exposure to AgNPs, finding a notable increase in the expression of genes related to lipopolysaccharide biosynthesis (*waaA*, *waaB*, *waaO*, *waaP*, *waaQ*, *waaR*, *waaS*, and *waaY*). This indicates that bacteria react to AgNPs-induced membrane stress by boosting lipopolysaccharide production. Moreover, lipopolysaccharide has been shown to shield cells by binding to cationic substances, thus preventing direct interaction between cationic toxins (like Ag^+^) and the bacterial outer membrane [216]. Petrova and colleagues [217] studied how an increase in copper content affects the activity of *Azospirillum baldaniorum* on wheat seedling roots and the physicochemical properties of synthetic lipopolysaccharide. Their results showed that adding 0.001/0.5 mM Cu^2+^ to the medium resulted in different ratios of LPS content to biomass in Sp245 biofilms and its mutants (0.7/2.4, 4.8/6.0, and 1.1/2.8), indicating higher polysaccharide antigen production under excessive Cu^2+^ conditions.

## Future Perspectives

In recent years, the global biosurfactant market has experienced significant growth due to its wide range of functional properties. Biosurfactants are used in various industries such as food processing, oil exploration, medical device disinfection, cosmetics, and environmental protection. However, the production of biosurfactants on an industrial scale is heavily influenced by factors such as microbial strains, substrate composition, fermentation processes, and other conditions, which pose challenges for scaling up production. To address these challenges and facilitate the commercialization and industrial use of biosurfactants, several strategies can be considered. These include using renewable, easily accessible, and cost-effective substrates, as well as optimizing fermentation parameters and processes. Additionally, genetic engineering techniques aimed at improving biosurfactant production in microbial strains show promise for industrial applications. In conclusion, future efforts can focus on the following key areas:

(1) Exploring the key genes responsible for biosurfactant production pathways controlled by metal ions through transcriptomic analysis.

(2) Delving into the generation of biosurfactants and other metabolites in strains influenced by metal ions using metabolomics techniques.

(3) Investigating the synthesis of biosurfactants and other metabolites in strains impacted by metal ions with the help of metabolomics techniques.

(4) Formulating high-producing biosurfactant strains and engineered strains.

## Conclusion

This review examines the various ways in which biosurfactants are synthesized. Glycolipid biosurfactants typically undergo fatty acid chain elongation through the chain-shortening pathway for their lipophilic component, while the hydrophilic glycosyl groups are produced through the TCA cycle or sugar metabolism. Lipopeptide biosurfactants have peptide rings that are synthesized by NRPS, and phospholipid biosurfactants can have structural variations through changes in the phosphate group composition. Polymeric biosurfactants are made up of repeated units of lipids and polysaccharides.

The review also discusses the importance of metal ions in the growth medium. Metal ions can enhance strain growth and development, leading to increased biosurfactant yields. Metal ions also have the ability to activate genes involved in biosurfactant synthesis, affecting both the yield and structure of the biosurfactants. Therefore, studying biosurfactant synthesis pathways and their regulation by metal ions is crucial for improving the production of high-yield biosurfactants and advancing their industrial applications.

## Figures and Tables

**Table 1:** The peptide sequence of Iturins.

Isoforms	Peptide sequence							References
	L1	D2	D3	L4	L5	D6	L7	
Iturin A	Asn	Tyr	Asn	Gln	Pro	Asn	Ser	[148]
Iturin C	Asp	Tyr	Asn	Gln	Pro	Asn	Ser	[148]
Iturin D	Asp	Tyr	Asp	Gln	Pro	Asn	Ser	[148]
Bacillomycin D	Asp	Tyr	Gln	Pro	Glu	Ser	Thr	[139]
Bacillopeptin	Asn	Tyr	Asn	Gln	Pro	Ser	Thr	[149]
Mycosubtilin	Asn	Tyr	Asn	Gln	Pro	Ser	Asn	[141]

**Table 2:** Effects of metal ions on glycolipids biosynthesis.

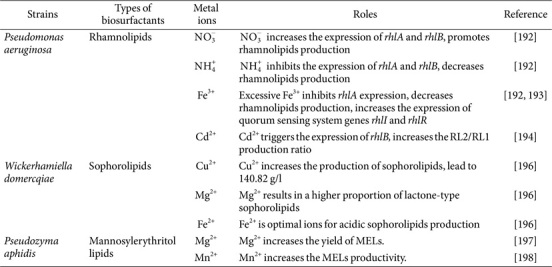

**Table 3:** Effects of metal ions on lipopeptides biosynthesis.

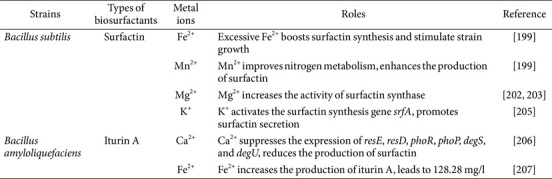

**Table 4:** Effects of metal ions on phospholipids biosynthesis.

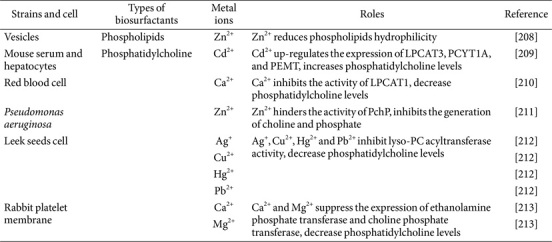

**Table 5:** Effects of metal ions on polymeric biosurfactants synthesis.